# Hypolipidemic and Hepatoprotective Effects of Polysaccharides Extracted from *Liriope spicata* Var. *Prolifera* in C57BL/6J Mice with High-Fat Diet-Induced Hyperlipidemia

**DOI:** 10.1155/2020/8013189

**Published:** 2020-12-10

**Authors:** Yi-Hui Liu, Zhi-Nan Xiang, Chen Chen, Luo-Sheng Wan, Jia-Chun Chen

**Affiliations:** ^1^Department of Pharmacy, Union Hospital, Tongji Medical College, Huazhong University of Science and Technology, Wuhan 430022, China; ^2^Hubei Key Laboratory of Natural Medicinal Chemistry and Resource Evaluation, Pharmacy Department of Tongji Medical School, Huazhong University of Science and Technology, Wuhan 430030, China

## Abstract

In this study, C57BL/6J mice with high-fat diet- (HFD-) induced hyperlipidemia were treated with total *Liriope spicata* var. *prolifera* polysaccharides (TLSP: 200, 400, and 800 mg/kg body weight), simvastatin (3 mg/kg body weight), or saline for 8 weeks, respectively. The results showed that TLSP had strong lipid-lowering and hepatoprotective effects on C57BL/6J mice with HFD-induced hyperlipidemia. TLSP administration significantly reduced serum total cholesterol (TC), triglyceride (TG), and low-density lipoprotein cholesterol (LDL-C) levels and downregulated the expressions of peroxisome proliferator-activated receptor (PPAR)*γ* and fatty acid synthase (FAS) in the adipose and liver tissues of the mice. TLSP exerted hypolipidemic and hepatoprotective effects by activating lipid/bile acid metabolism via the FXH-SHP/CYP7A1 and SEBP-1c/FAC/ACC signaling pathways. Thus, TLPS is a promising natural polymer with hepatoprotective and hypolipidemic properties.

## 1. Introduction

Hyperlipidemia is characterized by an elevation in the levels of total cholesterol (TC), triglycerides (TGs), and low-density lipoprotein cholesterol (LDL-C) along with a decline in the level of high-density lipoprotein cholesterol (HDL-C). Between 1980 and 2014, the prevalence of hyperlipidemia has increased dramatically, especially in fast-growing economies like China [1]. Imbalance between energy intake and consumption and obesity caused by unhealthy lifestyles will lead to hyperlipidemia, insulin resistance, and leptin resistance [2, 3]. These disorders of metabolism are major risk factors for atherosclerosis, type 2 diabetes mellitus (T2DM), nonalcoholic fatty liver disease, and other fatal diseases [2–4]. Various pharmaceutical agents have been applied in the treatment of hyperlipidemia, including statins, ezetimibe, and fibrates. Such interventions have shown promising effectiveness in lipid management but can also cause many side effects like liver injury, muscle aches, and digestive dysfunction. Therefore, researchers have turned their attention to herbal medicines to seek for safer and more cost-effective alternatives for the treatment of hyperlipidemia.

**Figure 1:**
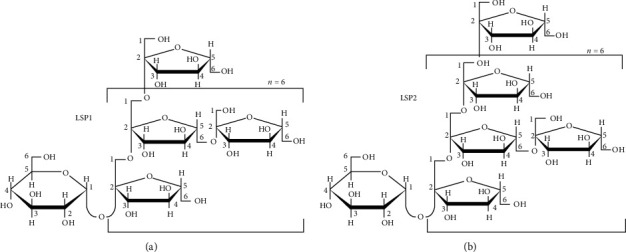
The structure of TLSP, including (a) LSP1 and (b) LSP2, extracted from LS.

*Liriope spicata* var. *prolifera* (Liliaceae) is indigenous to Hubei Province, China. Its tuberous root, recorded as Liriopes Radix in the Pharmacopoeia of the People's Republic of China, is frequently used in traditional Chinese medicine as the treatment of T2DM and inflammatory and cardiovascular diseases [5]. We have previously demonstrated that the total polysaccharide extract isolated from this tuberous root is composed of two fructans, named LSP1 and LSP2 (Figure 1), which have an average molecular weight of 3.20 and 4.29 kDa, respectively. Both fructans have the same backbone structure consisting of *β*-(1-2)-fructosyl residues that branches at O-6 with fructosyl residues and terminates with a glucosyl residue and a fructosyl residue [6, 7]. In our previous studies, total *Liriope spicata* var. *prolifera* polysaccharides (TLSP) exhibited promising effects on both glycemic control and lipid management in diabetic rodents' model through the elevation of insulin signal transduction [8–10]. Considering the antidiabetic effects of TLSP, we hypothesized that TLSP might also be a satisfactory alternative medicine for the treatment of hyperlipidemia. However, *in vivo* experiments are required to validate this hypothesis.

Therefore, in the present study, we evaluated the hypolipidemic effects of TLSP in C57BL/6J mice with high-fat diet- (HFD-) induced hyperlipidemia, and the mRNA expressions of genes related to cholesterol, bile acid, and fatty acid metabolism are also measured to explore the mechanisms underlying the effects of TLSP on lipid metabolism.

## 2. Materials and Methods

### 2.1. Chemicals and Diet

TLSP was extracted from the tuberous root of *Liriope spicata* var. *prolifera* harvested in Hubei, China. The TLSP (LSP1 + LSP2) preparation was made according to the method described in our previous studies [6, 8]. High-Fat Diet (HFD: D12492; including protein 26.2 g/100 g, carbohydrate 26.3 g/100 g, and fat 34.9 g/100 g) was purchased from Research Diets Inc. (New Brunswick, NJ, USA). The other reagents used in this experiment were purchased from local chemical suppliers in China.

### 2.2. Animal Experiments

Male C57BL/6J mice (4 weeks old) were purchased from the Beijing Vital River Laboratory Animal Technology Co. Ltd. (Beijing, China). All animals were housed under standard conditions (12 h light and dark cycle, 22°C ± 2°C, and 55% relative humidity), with free access to water and standard chow (protocol no. SPF2013100). After a 1-week acclimatization period, all mice were weighed and randomly divided into 6 groups of 8 mice each. The mice in the normal control (NC) group received standard chow. The mice in the HFD group received HFD and were orally administered a 0.1% (w/v) aqueous solution of sodium carboxymethyl cellulose (vehicle). The mice in three of the remaining four groups were given HFD and orally administered a low, medium, or high dose of TLSP (200, 400, and 800 mg/kg body weight); these groups were denoted as the TLSP(L), TLSP(M), and TLSP(H) groups, respectively. The mice in the last group, termed the SIM group, also received HFD and were orally administered simvastatin (3 mg/kg body weight).

The experiment lasted for 8 weeks. Body weight and food intake were measured weekly. The study was approved by the Institutional Animal Care and Use Committee of Tongji Medical College, Huazhong University of Science and Technology, and was in accordance with the Regulations for the Administration of Affairs Concerning Experimental Animals in Hubei Province, China.

After the 8-week experimental period, blood samples were collected from the retrobulbar vein of each mouse under anesthesia and stored at −80°C. The liver and adipose tissues (epididymal and perirenal adipose tissues) were collected, weighed, homogenized, and stored at −80°C until RNA extraction and further analysis.

### 2.3. Biochemical Measurements

Serum TC, TG, LDL-C, and HDL-C content was measured using the previously described methods [11, 12]. The serum total bile acid content was measured using an enzymatic method. Total bile acids were extracted using 75% ethanol at 50°C for 2 h, and their concentration was determined using a total bile acid assay kit (BioQuant, Heidelberg, Germany). The total antioxidant capacity (T-AOC), and levels of malondialdehyde (MDA), superoxide dismutase (SOD), and glutathione peroxidase (GSH-PX) were determined using assay kits, according to the manufacturer's directions (Beijing Bios Biotechnology Co. Ltd., Beijing, China). Aspartate transaminase (AST) and alanine transaminase (ALT) activities were measured in serum by kits.TC and TGs were extracted from the liver tissue samples of the mice and measured by using commercial kits (Nanjing Jiancheng Bioengineering Institute, Nanjing, China).

### 2.4. Histological Analysis and Morphometry

Liver and adipose tissue samples of the mice were embedded in a tissue-freezing medium (Tissue-Tek OCT compound; Miles Inc.) and frozen in liquid nitrogen immediately after being harvested. The embedded samples were cut into 10 *μ*m sections and stained with hematoxylin and eosin for histological examination under an optical microscope (Nikon Instruments Co. Ltd., Japan) [13].

### 2.5. Real-Time Reverse Transcriptase-Polymerase Chain Reaction

Total RNA was extracted from the liver and adipose tissue samples of the mice by using the TRIzol reagent, and cDNA was synthesized using a GeneAmpRNA PCR kit (Invitrogen Life Technologies, Carlsbad, CA, USA) according to the manufacturer's instructions. The mRNA expression levels of selected genes were determined using quantitative real-time reverse transcriptase-polymerase chain reaction (qRT-PCR) assays performed on an ABI StepOne Plus Real-Time PCR system (Applied Biosystems, Foster City, CA, USA). The PCR parameters were as follows: one cycle at 95°C for 5 min, followed by 40 cycles at 95°C for 15 s and 60°C for 1 min. The sequences of the primers used for the qRT-PCR assays are shown in Table 1. The mRNA levels of all genes examined were normalized using *β*-actin (*GAPDH*) expression as the internal control. The relative expressions of the mRNAs were calculated as previously described [14].

### 2.6. Statistical Analysis

All data were expressed as mean ± SD. We used *t*-tests and one-way analysis of variance to determine significant differences in group means. *P* < 0.05 was considered statistically significant.

## 3. Results

### 3.1. Effects of TLSP on Body Weight, Food Intake, and Food Efficiency Ratio

**Figure 2:**
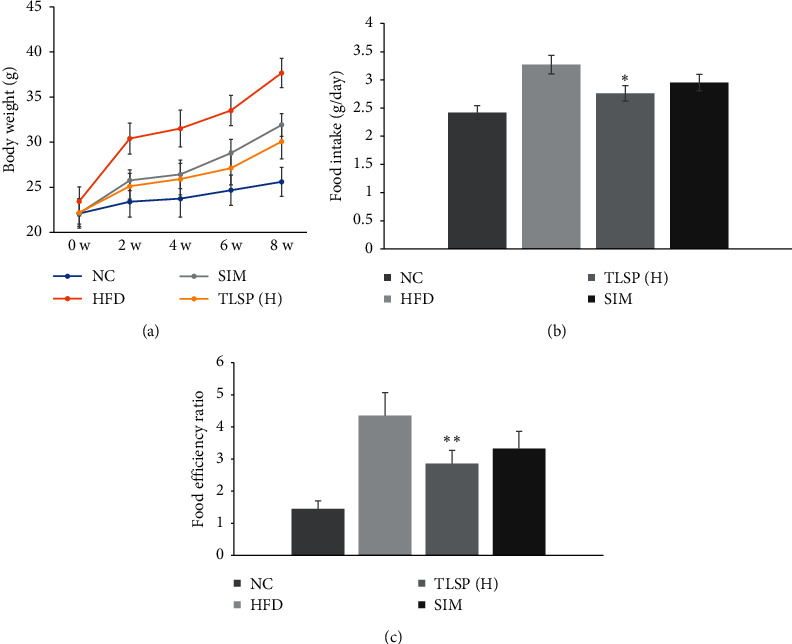
Effect of TLSP on body weight (a), food intake (b), and FER (food efficiency ratio) (c) in mice with HFD-induced hyperlipidemia. TLSP: total LS polysaccharides from tuberous root of *Liriope spicata* var. *prolifera* (Liliaceae); NC: normal control; HFD: high-fat diet; SIM: simvastatin; and TLSP(H): high-dose TLSP.

The changes in body weight and food intake from 0 to 8 weeks are shown in Figures 2(a) and 2(b), respectively. Mice in the HFD group showed a rapid increase in body weight as compared with mice in the NC group (*P* < 0.01). TLSP treatment decreased the HFD-induced gain in body weight in a dose- and time-dependent manner but did not significantly affect the food intake of C57BL/6J mice, as compared with the HFD group. These data also suggested that TLSP reduced the food efficiency ratio (FER) induced by the HFD.

### 3.2. Effects of TLSP on Plasma Lipid Levels

**Figure 3:**
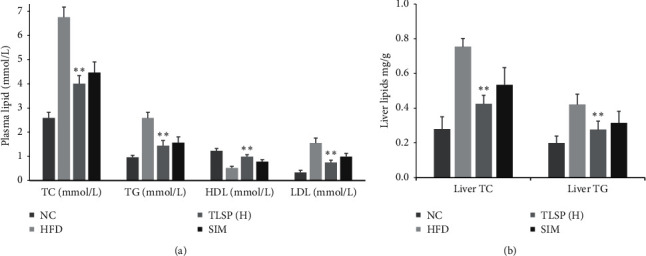
Effect of TLSP on plasma lipid (a) and liver lipid (b) levels in mice with HFD-induced hyperlipidemia. NC: normal control; HFD: high-fat diet; SIM: simvastatin; TLSP(H): high-dose total polysaccharides from tuberous root of *Liriope spicata* var. *prolifera* (Liliaceae); TC: total cholesterol; TG: triglycerides; HDL-C: high-density lipoprotein cholesterol; and LDL-C: low-density lipoprotein cholesterol.

The plasma TC level was 40.8% lower in the TLSP(H) group than in the HFD group (*P* < 0.05; Figure 3(a)). We also observed significant reductions in TC levels in the TLSP(M) and TLSP(L) groups as compared to the HFD group. The TG levels were also markedly lower in the TLSP groups than in the HFD group. Similarly, plasma LDL-C levels were lower in the TLSP groups than in the HFD group and were especially lower in the TLSP(H) group (52.3% of the level in the HFD group). The HDL-C level was significantly higher in the TLSP(H) group than in the HFD group (*P* < 0.05). Moreover, the TG and LDL-C levels in the TLSP(H) group were remarkably lower than those in the SIM group.

The liver TC and TG levels were also significantly lower in the TLSP groups and the SIM group than in the HFD group (*P* < 0.05; Figure 3(b)). After 8 weeks of administration, the hepatic levels of LDL-C were decreased in the TLSP and SIM groups (*P* < 0.01).

### 3.3. Effects of TLSP on Biochemical Parameters

**Figure 4:**
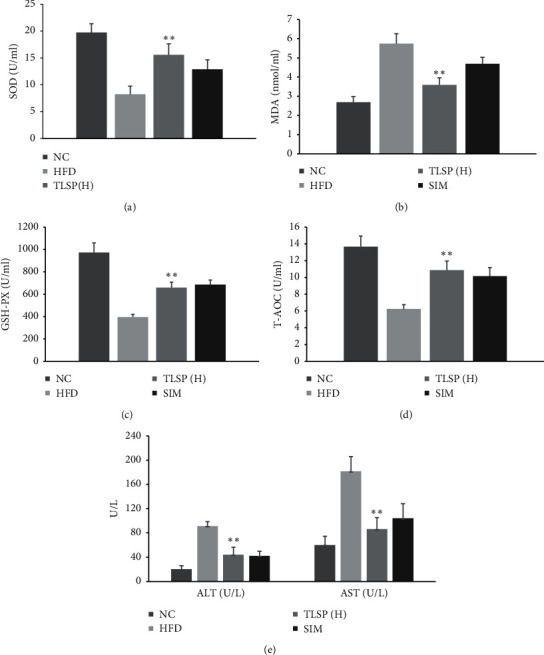
Effects of TLSP on serum biochemical parameters. (a) SOD, (b) MDA, (c) GSH-PX, (d) T-AOC, and (e) ALT, AST. Data are expressed as mean ± SD (*n* = 7). ^*∗*^*P* < 0.05, ^*∗∗*^*P* < 0.01 versus the HFD group. NC: normal control; HFD: high-fat diet; SIM: simvastatin; TLSP(H): high-dose total polysaccharides from tuberous root of *Liriope spicata* var. *prolifera* (Liliaceae); SOD: superoxide dismutase; MDA: malondialdehyde; GSH-PX: glutathione peroxidase; T-AOC: total antioxidant capacity; ALT: alanine transaminase; and AST: aspartate transaminase (AST).

To explore the possible mechanisms underlying the hepatoprotective and antioxidant properties of TLSP, we measured the ALT, AST, T-AOC, SOD, GSH-Px, and MDA levels. As shown in Figure 4, the T-AOC, and SOD, and GSH-Px levels were remarkably decreased in the HFD group as compared with the NC group during the 8 weeks of administration. The MDA content, ALT, and AST level were much higher in the HFD group than in the NC group as shown in Figures 4(a)–4(e)*P* < 0.05). In the TLSP(H) group, the T-AOC and SOD and GSH-PX activities increased significantly by 74.11%, 89.17%, and 66.15%, respectively, while the MDA content ALT and AST level reduced by 37.33%, 90.63%, and 181.0% as compared with the HFD group (*P* < 0.05).

### 3.4. Effects of TLSP on Bile Acids

**Figure 5:**
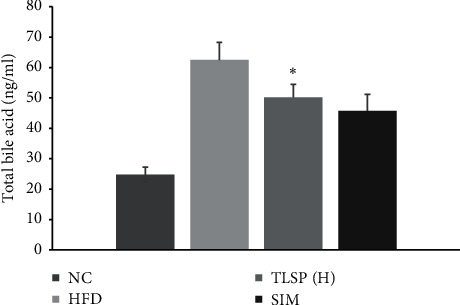
Effects of TLSP on serum total bile acids. Data are presented as mean ± SD (*n* = 7). ^*∗*^*P* < 0.05, ^*∗∗*^*P* < 0.01 versus the HFD group. NC: normal control; HFD: high-fat diet; SIM: simvastatin; and TLSP(H): high-dose total polysaccharides from tuberous root of *Liriope spicata* var. *prolifera* (Liliaceae).

The total bile acids in the TLSP and SIM groups were increased as compared with the HFD group (*P* < 0.01; Figure 5). In the TLSP(H) and SIM groups, the total bile acid content was decreased by 21% and 15.9%, respectively, as compared with the NC group (*P* < 0.05). These findings indicated that TLSP could reduce the total bile acid levels, especially at high doses.

### 3.5. Effects of TLSP on Liver and Adipose Tissue Histology

**Figure 6:**
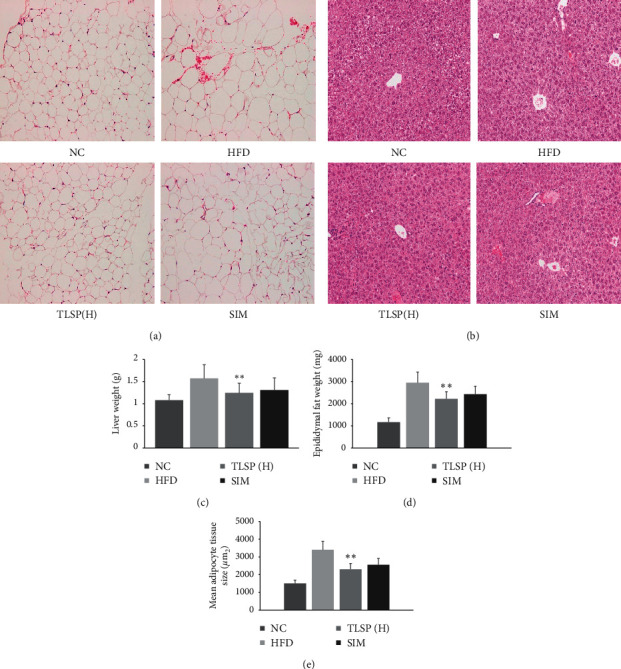
(a) Effects of TLSP on histopathological changes in liver sections of HFD mice. (b) Effect of TLSP on fat accumulation in epididymal fat pads of HFD mice, effect of TLSP on liver weight (c), epididymal fat weight (d), and mean adipocyte tissue size (*μ*m^2^) score (e) (hematoxylin and eosin-stained sections photographed at 400× magnification). NC: normal control; HFD: high-fat diet; SIM: simvastatin; and TLSP(H): and high-dose total polysaccharides from tuberous root of *Liriope spicata* var. *prolifera* (Liliaceae).

Since the liver is the center of fatty acid synthesis and lipid metabolism (Tessari, Coracina, Cosma, and Tiengo, 2009), we evaluated hematoxylin and eosin-stained sections of liver tissues from all groups for fat accumulation and morphology. Increase in liver weight, fat accumulation, and hepatic steatosis was observed in the HFD group (Figures 6(a) and 6(c)). The amount of fat accumulated in the liver was obviously lower in the TLSP and SIM groups than in the HFD group. Moreover, hepatic fat accumulation did not differ between the SIM and TLSP groups.

Hematoxylin and eosin-stained sections of epididymal adipose tissues were also evaluated. Compared with the NC group, the HFD group showed more fat accumulation and severe hypertrophy in most epididymal adipose tissues (Figure 6(b)). The adipocyte size was remarkably lower in the TLSP groups than in the HFD group (*P* < 0.05; Figures 6(d) and 6(e)). In particular, adipocyte hypertrophy and adipocyte size were significantly ameliorated in the TLSP(H) group as compared to the HFD group. However, the adipocyte size was significantly smaller in the SIM group than in the TLSP groups (*P* < 0.05; Figure 6(e)).

### 3.6. Effects of TLSP on Gene Expression in Liver Tissue

**Figure 7:**
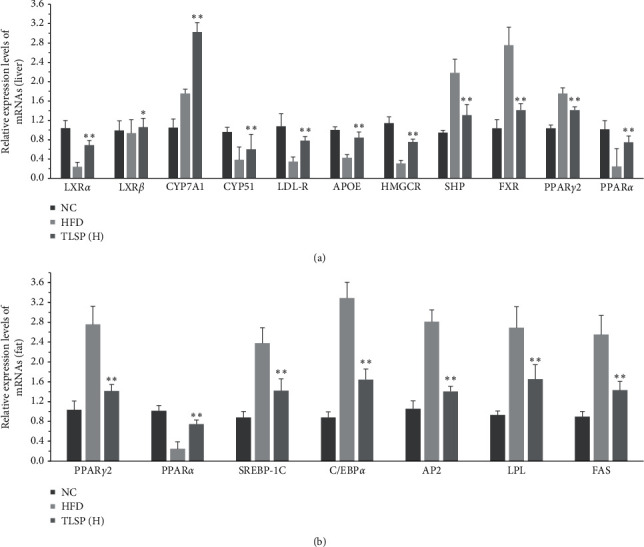
Effects of TLSP on mRNA expressions of genes involved in the peroxisome proliferator-activated receptor (PPAR) signaling pathway and genes related to bile acid and lipid metabolism in the liver tissue (a) and epididymal adipose tissue (b). Data are presented as mean ± SD (*n* = 7). ^*∗*^*P* < 0.05, ^*∗∗*^*P* < 0.01 versus the HFD group. NC: normal control; HFD: high-fat diet; SIM: simvastatin; and TLSP(H): high-dose total polysaccharides from tuberous root of *Liriope spicata* var. *prolifera* (Liliaceae).

To reveal the molecular mechanisms underlying the lipid-lowering effects of TLSP, we determined the hepatic mRNA expression of genes related to cholesterol and bile acid metabolism by using qRT-PCR. The mRNA expressions of Liver X receptor alpha (LXR*α*), LXR*β*, SHP, farnesoid X receptor (FXR), CYP7A1, CYP51, HMGCR, APOE, LDL-R, and peroxisome proliferator-activated receptor *α* (PPAR*α*) were 1.87-, 1.13-, 1.32-, 1.66-, 1.89-, 0.58-, 1.45-, 0.98-, 1.26-, and 2.13-fold greater, while the mRNA expressions of ABCG5 and PPAR*γ*2 were 0.36- and 0.19-fold lower, respectively, in the TLSP(H) group as compared to the HFD group (Figure 7(a)). Remarkable activation of the LXR/FXR/SHP/CYP7A1 signaling pathway was observed in the TLSP(H) group (*P* < 0.05; Figure 7(b)). We found that the HFD-induced increase in the expression of PPAR*γ*2 was improved by TLSP administration.

The above data indicated that TLSP could regulate adipocyte differentiation and lipid balance via the PPAR signaling pathway. Specifically, TLSP treatment resulted in a reduction in PPAR*γ* expression and elevations in PPAR*α*, SHP, FXR, LXR*α*, LXR*β*, CYP7A1, CYP51, LDL-R, APOE, and HMGCR gene expressions. In brief, the relative expressions of the genes involved in lipid/fatty acid metabolism were significantly altered in all three TLSP groups, with the greatest alteration being observed in the TLSP(H) group.

### 3.7. Effects of TLSP on Gene Expression in Adipose Tissue

The mRNA expressions of genes related to lipid metabolism in adipose tissues were also measured by qRT-PCR. Consistent with the above results, we found a remarkable reduction in the PPAR*γ*2 expression level in the TLSP(H) group as compared with the HFD group (*P* < 0.05). The mRNA expressions of SREBP-1c, FAS, ACC, and AP2, which are involved in lipid and fatty acid synthesis, were downregulated in the TLSP groups, while the PPAR*α* expression was upregulated in the TLSP groups as compared with the HFD group (Figure 7(b)). The mRNA expression levels of PPAR*γ*, SREBP-1c, C/EBP*α*, FAS, AP2, and ACC were significantly reduced (0.48-, 0.40-, 0.49-, 0.44-, 0.50-, and 0.39-fold lower), while the PPAR*α* expression was increased (2.05-fold greater) in the TLSP(H) group as compared to the HFD group (Figure 7(b)). Additionally, the changes in the PPAR*γ*/SREBP-1c/ C/EBP*α*/AP2/ACC/FAS signaling pathway were greater in the TLSP groups than in the SIM group (*P* < 0.05). Treatment with TLSP could reduce the expression levels of these important factors to close to normal levels (*P* < 0.05 compared with the HFD group).

## 4. Discussion

Therapy for hyperlipidemia has been extensively researched, due to the strong and compelling evidence showing that good lipid management can prevent cardiovascular disease [15, 16]. Various polysaccharides are currently used as adjuncts to conventional therapies in the treatment of metabolic disorders, including hyperlipidemia, and, thus, there is great potential for the development of candidate drugs [17–20]. Our previous studies have demonstrated that TLSP contributes to glycemic control and lipid management in diabetic rodents [6–10]. In this study, we further investigated the lipid-lowering effects of TLSP in HFD-fed C57BL6J mice. We found that, compared with the HFD group, the TLSP groups had lower food intakes, reduced body weight gain, and decreased plasma lipid levels. In addition, TLSP treatment resulted in a remarkable reduction in the sizes of both hepatocytes and adipocytes.

An unhealthy diet and lack of exercise are known to cause overweight and even obesity in the long term [21]. Overweight or obese patients are in a state of metabolic disorder, with serum lipid dysfunction and excessive fat accumulation [21]. PPARs are transcription factors that are mainly expressed in adipocytes. PPARs play key roles in the regulation of energy homeostasis, lipid metabolism, and inflammation response [22–24]. HFD activates the expression of multiple genes related to fatty/bile acid metabolism and regulates PPAR activation in adipose tissue [25–27]. Moreover, SREBP-1c regulates PPAR*γ* activation in adipocytes and promotes the expression of a number of genes involved in fatty acid synthesis [27–29]. Increased SREBP-1c expression leads to the activation of lipogenic genes like FAS and ACC [29]. Activated FAS and ACC perform key roles in fatty acid conversion and triacylglycerol synthesis [28]. A high FAS level is closely related to high levels of glucose and cholesterol and is usually observed in obese individuals [30]. AP2 integrates inflammatory and metabolic responses [31, 32]. As the main transcription factors of lipogenesis, activated PPAR*γ*/SREBP1c activate downstream target genes of fatty acid synthase, like FAS, ACC, and AP2, which accelerate TG synthesis and lipid accumulation [33]. Thus, the abnormal expression of key genes leads to dyslipidemia and insulin resistance, further deteriorating glycemic control and lipid management. Therefore, to explore the possible mechanisms underlying the role of TLSP in lipid management, we performed qRT-PCR assays to evaluate the mRNA expressions of the above genes in the hepatic and adipose tissues of mice with HFD-induced hyperlipidemia. As expected, the mRNA expression of PPAR*γ*2 was significantly suppressed while that of PPAR*α* was remarkably enhanced in the TLSP(H) group as compared with the HFD group. In addition, the mRNA expression levels of SREBP-1c, C/EBP*α*, FAS, ACC, and AP2 were significantly lower in the TLSP(H) group than in the HFD group (Figure 7(b)). These results suggest that TLSP exerts lipid-lowering and antiobesity effects by inhibiting PPAR*γ*2 and the SREBP-1 pathway and may be an efficacious therapeutic agent to prevent lipid metabolic disorder and regulate adipocyte differentiation and proliferation. Consistent with this, TLSP has been reported to block diseases such as hyperlipidemia, hyperglycemia, metabolic syndrome, atherosclerosis, and cardiovascular diseases [2–4, 34].

The liver plays a key role in the regulation of cholesterol and bile acid metabolism. Our previous studies indicated that the cholesterol-lowering properties of natural products are usually associated with the modulation of bile acids. To further explore the mechanism underlying the lipid- and cholesterol-regulating activities of TLSP, we analyzed the mRNA expressions of relevant genes in the liver tissues of HFD mice. The nuclear receptors FXR and LXR are rate-controlling genes in bile acid metabolism [35–37]. FXR affects bile acids by regulating the expression of genes involved in bile acid and cholesterol homeostasis (CYP7A1, CYP51, APOE, and SHP) [37–39]. CYP7A1 is also a rate-limiting gene in the bile acid synthetic pathway in the liver [40, 41], and it is regulated by the nuclear receptors FXR, SHP, and LXR [37]. Studies have indicated that the gene expression and activity of CYP7A1 can be elevated by a high-cholesterol diet [37, 40]. We hypothesize that TLSP regulates key signaling pathways involved in lipid metabolism and indirectly activates genes expressed in the liver and adipose tissues. The results of qRT-PCR are consistent with this hypothesis. TLSP may upregulate the hepatic mRNA expression of CYP7A1 and LDL-R by accelerating cholesterol efflux from the circulation and promoting bile acid synthesis [42]. The expressions of FXR, SHP, LXR*α*, LXR*β*, CYP7A1, CYP51, and ApoE were downregulated in the NC group, and TLSP treatment reversed their expression levels (Figure 7(a)), which suggests that TLSP ameliorated hyperlipidemia and accelerated cholesterol and bile acid metabolism via the LXR/FXR-SHP/CYP7A1 signaling pathway.

As a key enzyme involved in cholesterol metabolism, HMGCR contributes to an increase in the levels of cholesterol and LDL-R [42]. Research has indicated that an increase in LDL-C concentration is a major risk factor for atherosclerosis and coronary heart disease [43]. HMGCR inhibitors (e.g., simvastatin) increase the gene expression of LDL-R, which accelerates the metabolism of plasma TC and LDL-C [42–44]. The reduced TC and LDL-C levels in the serum in our study were partly a result of the upregulation of HMGCR and LDL-R mRNA expressions by TLSP supplementation. The significant elevation of LDL-R and HMGCR mRNA expression in the TLSP groups indicated that TLSP could promote the clearance of plasma TC and LDL-C levels, which serves as an important physiological route for the recycling of bile acids and regulation of lipid homeostasis. Moreover, we also observed the downregulation of hepatic ABCG5 mRNA expression by TLSP treatment; ABCG5 retains hepatic cholesterol by controlling cholesterol efflux to bile acid synthesis. The elevation of CYP7A1 mRNA expression might also have partly contributed to bile acid metabolism [45], implying that the lipid-lowering effects of TLSP were probably the result of increased bile acid metabolism and reduced cholesterol content [45–48].

Consistent with the above results, the histological examination of liver tissues showed that the progression of steatosis and hepatocyte hypertrophy was ameliorated after TLSP treatment, which can be partly explained by the adjustment of lipid balance and the hypolipidemic effects of TLSP. The change trend of ALT and AST is also consistent with the results of promoting cholesterol excretion and liver protection activity. Thus, TLSP had important effects on lipid deposition, adipocyte differentiation, and cholesterol efflux, and hepatoprotection.

The results of the analysis of the biochemical parameters of antioxidant activity were consistent with the above outcomes. Oxidative stress is mainly caused by an increase in the production of reactive oxygen species and plays a key role in the development of hyperlipidemia [49, 50]. Indeed, oxidative damage is the main cause of hyperlipidemia and its related diseases, including type 2 diabetes, metabolic syndrome, nonalcoholic fatty liver, obesity, atherosclerosis, and other cardiovascular diseases [51, 52]. Antioxidant enzymes and related parameters, including SOD, GSH-PX, MDA, and T-AOC, are directly involved in the defense against oxidative damage, and the risk of hyperlipidemia and atherosclerosis [53, 54]. Our results showed that TLSP could ameliorate oxidative stress by promoting the activities of the antioxidant enzymes SOD and GSH-PX, and by decreasing MDA content. This indicates that the hepatoprotective and lipid-lowering effects of TLSP in C57BL/6J mice with HFD-induced hyperlipidemia might be partially attributable to an increase in antioxidant enzyme activities [53, 54].

The present study has demonstrated that TLSP indeed reduced body weight gain food intake and food efficiency ratio; ameliorated lipid accumulation and hepatic steatosis; and alleviated the progression of hyperlipidemia in mice with HFD-induced hyperlipidemia. The lipid-lowering effects of TLSP appeared to be accompanied by and linked to the regulation of bile acid and fatty acid metabolism. We have demonstrated that the improvements in lipid metabolism after TLSP treatment were attributable to the upregulation of the LXR/FXR-SHP/CYP7A1 signaling pathway. FXR-SHP inhibits SREBP-1c [28, 29, 35], and the activation of FXR-SHP/CYP7A1 facilitated the efflux of lipids by downregulating the SREBP-1c/FAS/ACC pathway and suppressing its downstream target genes, which are involved in fatty acid synthesis, thereby alleviating the progression of dyslipidemia [28–30, 32]. Moreover, TLSP reduced plasma TC levels and regulated lipid balance without causing any adverse reactions. Therefore, TLSP supplementation could reduce plasma TC levels by promoting fatty acid and bile acid suppression, activating antioxidant enzyme activities, and balancing lipid homeostasis.

There are several limitations to our current study. Although TLSP could reduce lipids levels via regulating bile acid metabolism-related pathways, we did not determine the effects of TLSP on gut microbiota. The possible antiobesity effects of TLSP have not been investigated. Thus, further research is needed.

## 5. Conclusion

In summary, TLSP showed strong lipid-lowering and hepatoprotective activities in C57BL/6J mice with HFD-induced hyperlipidemia. This study suggests that TLSP treatment has profound potential as a therapeutic agent that prevents the development of hyperlipidemia, obesity, atherosclerosis, and cardiovascular disease.

## Figures and Tables

**Table 1:** Sequences of primers used in real-time RT-PCR.

Gene	Forward primer	Reverse primer
GAPDH	ATGGGTGTGAACCACGAGA	CAGGGATGATGTTCTGGGCA
PPAR*α*	AGACAAAGAGGCAGAGGTCC	CGATCAGCATCCCGTCTTTG
PPAR*γ*2	AGGGCGATCTTGACAGGAAA	CGAAACTGGCACCCTTGAAA
LXR*α*	GCATGATCGAGAAGCTGGTG	GTCTTCAGCAAGGCGATCTG
LXR*β*	GGCGTCCACCATTGAGAT	GCGATAAGCAAGGCATACTCT
CYP7A1	GGCATTTGGACACAGAAGCA	ATACATCCCTTCCGTGACCC
CYP51	AACTCAACGAGAAGGTGGCT	CTGAAACTTGGCAGAGGC
SREBP-1c	GTCAAAACCAGCCTCCCAAG	GTCCCCGTCCACAAAGAAAC
C/EBP*α*	GGTGATCAAACAAGAGCCCC	CGATCTGGAACTGCAAGTGG
FAS	CCTGCCTCTGGTGCTTGCT	GGGCCTCCTTGATATAATCCTT
ACC	GAATCTCCTGGTGACAATGCTTATT	GGTCTTGCTGAGTTGGGTTAGCT
AP2	TTTCCTTCAAACTGGGCGTG	CATTCCACCACCAGCTTGTC
ApoE	AGGCTAAGGACTTGTTTCGG	TGGTTGCTTTGCCACTCG
LDL-R	TTCAGTCCCAGGCAGCGTAT	TTGATCTTGGCGGGTGTT
HMGCR	CCTTGTTCACGCTCATAGTCG	CTTGCTCAATGTCCATGCTGA
SHP	AACCTGCCGTCCTTCTGC	CGCTGCTGGCTTCCTCTA
FXR	TTCCTCAAGTTCAGCCACAG	TCGCCTGAGTTCATAGATGC

## Data Availability

The datasets used and analyzed during this study are available from the corresponding authors upon reasonable request.
